# Supplementary material for: Deep Doubly Debiased Longitudinal Effect Estimation with ICE G-Computation
Source: KDD. Author manuscript; Available in PMC 2026 Oct 12. (PMC13654504; doi:10.1145/3770855.3818925)
Supplement: Supplementary file 1 [file NIHMS2214663-supplement-1.pdf]

A Proof

Lemma 3 (First- vs. second-order dependence in training targets). Assume bounded outcomes and positivity. Let $\hat{Q}_t^{\mathrm{ICE}}$ be the initial estimate from regular ICE G-computation, and let $\hat{Q}_t^{\mathrm{SDR}}$ be the initial estimate from SDR procedure. Throughout, we suppress function arguments when clear from context; e.g., Q_t^0 denotes $Q_t^0(A_t, H_t)$ and G_k^0 denotes $G_k^0(A_k \mid H_k)$ unless arguments are shown explicitly.

Then, for each $t = 1, \dots, \tau - 1$, the population regression errors admit the bounds

$$\|\hat{Q}_t^{\mathrm{ICE}} - Q_t^0\|_2 \;\le\; \xi_t^{\mathrm{ICE}} + \|\hat{Q}_{t+1}^{\mathrm{ICE}}(a_{t+1}, H_{t+1}) - Q_{t+1}^0(a_{t+1}, H_{t+1})\|_2,$$

$$\|\hat{Q}_t^{\mathrm{SDR}} - Q_t^0\|_2 \;\le\; \xi_t^{\mathrm{SDR}} + \sum_{k=t+1}^{\tau} C_{t,k}\, \|\hat{G}_k^{\mathrm{SDR}} - G_k^0\|_2\, \|\hat{Q}_k^{\mathrm{SDR}} - Q_k^{\dagger}\|_2,$$

for constants $C_{t,k}$ depending only on positivity and τ, where ξ_t^{ICE} and ξ_t^{SDR} denote the regression-to-target error at time t for corresponding estimator.

Proof. Consider a CF sequence $\boldsymbol{a} = (a_1, \dots, a_\tau)$. Recall the target regression at time t: $Q_t^0(A_t, H_t) := \mathbb{E}\big[\, Q_{t+1}^0(a_{t+1}, H_{t+1}) \mid A_t, H_t \,\big]$. Throughout, positivity means $G_k^0(H_k), \hat{G}_k^{\mathrm{SDR}}(H_k) \in [\delta, 1-\delta]$ a.s. for all $k \in [\tau]$, so all density ratios below are uniformly bounded.

Step 1: a generic decomposition. For any training target T_{t+1} (measurable w.r.t. (H_{t+1}, A_{t+1})), define its conditional mean: $m_t^T(A_t, H_t) := \mathbb{E}[T_{t+1} \mid A_t, H_t]$. Then by the triangle inequality,

$$\|\hat{Q}_t - Q_t^0\|_2 \le \underbrace{\|\hat{Q}_t - m_t^T\|_2}_{:=\xi_t} + \|m_t^T - Q_t^0\|_2. \tag{8}$$

We apply (8) to the ICE target and the SDR target separately.

Step 2: ICE target yields a first-order bias term. For ICE, the training target is $T_{t+1}^{\mathrm{ICE}} := \hat{Q}_{t+1}^{\mathrm{ICE}}(a_{t+1}, H_{t+1})$. Hence, $m_t^{\mathrm{ICE}} - Q_t^0 = \mathbb{E}\big[\hat{Q}_{t+1}^{\mathrm{ICE}}(a_{t+1}, H_{t+1}) - Q_{t+1}^0(a_{t+1}, H_{t+1}) \mid A_t, H_t\big]$. By Jensen (equivalently, $\|\mathbb{E}[U \mid A_t, H_t]\|_2 \le \|U\|_2$),

$$\|m_t^{\mathrm{ICE}} - Q_t^0\|_2 \le \|\hat{Q}_{t+1}^{\mathrm{ICE}}(a_{t+1}, H_{t+1}) - Q_{t+1}^0(a_{t+1}, H_{t+1})\|_2.$$

Combining this with (8) proves the ICE bound.

Step 3: SDR target yields a second-order bias term. For SDR, the training target is $T_{t+1}^{\mathrm{SDR}} := Q_{t+1}^{\dagger}(a_{t+1}, H_{t+1})$, where $Q^{\dagger}$ is constructed via (7) using nuisance estimates $(\hat{Q}^{\mathrm{SDR}}, \hat{G}^{\mathrm{SDR}})$. Let $m_t^{\mathrm{SDR}}(A_t, H_t) := \mathbb{E}[Q_{t+1}^{\dagger}(a_{t+1}, H_{t+1}) \mid A_t, H_t]$. We will bound $\|m_t^{\mathrm{SDR}} - Q_t^0\|_2$.

Define the true and estimated density ratios for the sequence $\boldsymbol{a}$:

$$w_k^0(A_k, H_k) := \frac{\mathbb{1}(A_k = a_k)}{A_k G_k^0(H_k) + (1 - A_k)(1 - G_k^0(H_k))},$$

$$\hat{w}_k(A_k, H_k) := \frac{\mathbb{1}(A_k = a_k)}{A_k \hat{G}_k^{\mathrm{SDR}}(H_k) + (1 - A_k)(1 - \hat{G}_k^{\mathrm{SDR}}(H_k))}.$$

Under positivity, $\|\hat{w}_k\|_\infty \vee \|w_k^0\|_\infty \le \delta^{-1}$ and

$$\|\hat{w}_k - w_k^0\|_2 \lesssim \|\hat{G}_k^{\mathrm{SDR}} - G_k^0\|_2. \tag{9}$$

Now consider the difference between the SDR pseudo-outcome and its population counterpart. Lemma 3 in Díaz et al. [12] implies that, for each fixed t,

$$m_t^{\mathrm{SDR}} - Q_t^0 = \sum_{k=t+1}^{\tau} \mathbb{E}\Big[W_{t,k}\, (\hat{w}_k - w_k^0)\, \big(\hat{Q}_k^{\mathrm{SDR}} - Q_k^{\dagger}\big) \,\Big|\, A_t, H_t\Big], \tag{10}$$

where $W_{t,k}$ is a product of (true and/or estimated) weight factors over times $t+1, \dots, k-1$. By positivity, $\|W_{t,k}\|_\infty \le C'_{t,k}$ for constants $C'_{t,k}$ depending only on (δ, τ).

Apply Jensen and Cauchy–Schwarz to (10):

$$\begin{aligned}
\|m_t^{\mathrm{SDR}} - Q_t^0\|_2 &\le \sum_{k=t+1}^{\tau} \Big\| E\Big[W_{t,k}\, (\hat{w}_k - w_k^0)\, (\hat{Q}_k^{\mathrm{SDR}} - Q_k^{\dagger}) \,\Big|\, A_t, H_t\Big]\Big\|_2 \\
&\le \sum_{k=t+1}^{\tau} \|W_{t,k}\|_\infty\, \|(\hat{w}_k - w_k^0)\, (\hat{Q}_k^{\mathrm{SDR}} - Q_k^{\dagger})\|_2 \\
&\le \sum_{k=t+1}^{\tau} C'_{t,k}\, \|\hat{w}_k - w_k^0\|_2\, \|\hat{Q}_k^{\mathrm{SDR}} - Q_k^{\dagger}\|_2.
\end{aligned}$$

Invoking (9) yields

$$\|m_t^{\mathrm{SDR}} - Q_t^0\|_2 \le \sum_{k=t+1}^{\tau} C_{t,k}\, \|\hat{G}_k^{\mathrm{SDR}} - G_k^0\|_2\, \|\hat{Q}_k^{\mathrm{SDR}} - Q_k^{\dagger}\|_2,$$

for constants $C_{t,k}$ depending only on (δ, τ). Finally, combine this bound with the generic decomposition (8) (with $T = T^{\mathrm{SDR}}$), where $\xi_t = \|\hat{Q}_t^{\mathrm{SDR}} - m_t^{\mathrm{SDR}}\|_2$, to obtain the SDR inequality in the theorem. □

B LTMLE

We adopted the one-step LTMLE algorithm [68] and added an L1 regularizer for the targeting update [4], as detailed in Algorithm 3.

C Implementation and Hyperparameter

Super-Learner-based G-computation and LTMLE are implemented using the `ltmle` R package, which constructs an optimally weighted ensemble of base learners via 3-fold cross-validation. Candidate learners include a generalized linear model, random forest, XGBoost, and a generalized additive model with regression splines. DeepACE and IGC-Net are implemented using the authors' original codebases [17, 23], while DeepLTMLE is implemented following the original paper. All deep learning models are trained for 100 epochs. For D^3-Net, the Polyak averaging coefficient is fixed at $\beta = 0.02$.

Table S1: Absolute bias (mean ± std) and RMSE of CAPO estimates at horizon $\tau = 10$.

Model	Limited Time-varying Confounding								Expanded Time-varying Confounding							
	Bias				RMSE				Bias				RMSE			
	CF 1	CF 2	CF 3	CF 4	CF 1	CF 2	CF 3	CF 4	CF 1	CF 2	CF 3	CF 4	CF 1	CF 2	CF 3	CF 4
G-comp (sl.)	3.03±1.19	3.81±1.52	2.81±1.47	4.33±1.39	3.25	4.09	3.16	4.53	1.93±0.80	3.34±0.92	1.82±0.48	3.10±1.21	2.08	3.46	1.88	3.32
LTM. (sl.)	2.80±0.89	0.63±0.41	**0.26±0.30**	3.65±1.39	2.93	0.75	0.39	3.89	2.01±0.71	0.58±0.67	0.21±0.19	3.41±0.87	2.14	0.87	0.28	3.51
D.ACE	0.93±0.16	0.52±0.40	0.84±0.52	0.78±0.26	0.95	0.65	0.98	0.82	0.98±0.11	0.69±0.37	0.78±0.46	0.87±0.11	0.99	0.77	0.90	0.88
D.LTMLE	0.72±0.56	0.59±0.49	0.61±0.34	1.35±1.11	0.91	0.76	0.70	1.73	0.51±0.45	0.37±0.42	0.41±0.26	0.78±0.52	0.68	0.55	0.48	0.93
IGC-Net	0.19±0.14	**0.14±0.10**	0.36±0.19	0.55±0.13	0.23	**0.17**	**0.41**	0.56	0.19±0.14	0.19±0.09	**0.36±0.21**	**0.48±0.09**	0.24	**0.21**	**0.42**	**0.49**
D^3-Net	**0.11±0.12**	0.19±0.27	0.51±0.10	**0.39±0.21**	**0.16**	0.32	0.52	**0.45**	**0.14±0.09**	0.16±0.17	0.56±0.10	0.61±0.32	**0.16**	0.23	0.57	0.68

Table S2: Absolute bias and RMSE of CAPO estimates at $\tau = 15$ under expanded confounding with additional covariates.

Model	Additional Covariates: numz = 10								Additional Covariates: numz = 15							
	Bias				RMSE				Bias				RMSE			
	CF 1	CF 2	CF 3	CF 4	CF 1	CF 2	CF 3	CF 4	CF 1	CF 2	CF 3	CF 4	CF 1	CF 2	CF 3	CF 4
D.ACE	0.91±0.16	0.40±0.09	0.69±0.24	0.54±0.34	0.93	0.40	0.73	0.63	0.96±0.18	0.36±0.09	0.74±0.27	0.29±0.20	0.97	0.37	0.78	0.35
D.LTMLE	0.75±0.88	1.00±0.95	0.55±0.24	0.43±0.43	1.14	1.36	0.60	0.60	1.03±1.04	0.92±0.71	0.55±0.34	0.32±0.26	1.45	1.15	0.64	0.40
IGC-Net	0.50±0.22	**0.15±0.11**	0.43±0.31	0.39±0.18	0.55	**0.18**	0.53	0.43	0.47±0.33	**0.21±0.15**	0.33±0.23	0.38±0.26	0.57	**0.25**	0.40	0.46
D^3-Net	**0.13±0.13**	0.19±0.12	**0.30±0.15**	**0.14±0.10**	**0.18**	0.22	**0.33**	**0.17**	**0.11±0.08**	0.23±0.22	**0.32±0.18**	**0.18±0.11**	**0.13**	0.32	**0.37**	**0.21**

Algorithm 3 LTMLE

1: **Input**: Dataset $\{O_i\}_{i=1}^n$; Initial estimates of Q_t and G_t for $t = 1, \dots, \tau$; L1 regularization coefficient: λ.
2: **Output**: CAPO estimate $\hat{\psi}_n = \mathbb{P}_n Q_{1,\epsilon_1}(a_1, H_1)$
3: Initialize $Q_{\tau+1} = Y$, $\epsilon_{\tau+1} = 0$
4: Initialize submodel $\text{logit}Q_{t,\epsilon_t} = \text{logit}Q_t + \epsilon_t$, and loss function

$$L_t(\epsilon_t) = \left(\prod_{s=1}^{t} \hat{w}_s(A_s, H_s)\right) \mathcal{L}_{bce}(Q_{t,\epsilon_t}(A_t, H_t), Q_{t+1,\epsilon_{t+1}}(a_{t+1}, H_{t+1})).$$

5: **for** $t = \tau$ to 1 **do**
6: $\quad \epsilon_t \leftarrow \arg\min_{\epsilon_t} \mathbb{P}_n L_t(\epsilon_t) + \lambda|\epsilon_t|$
7: **end for**

Hyperparameters are tuned at $\tau = 15$ using 800 trajectories for training and 200 for validation. Models are retrained on all 1000 trajectories using the selected hyperparameters. For $\tau = 10$ and $\tau = 20$, we reuse the hyperparameters selected at $\tau = 15$. We perform random search and select hyperparameters by minimizing the factual loss $L_{mse}(Q(A_\tau, H_\tau), Y) + \sum_{t=1}^{\tau} L_{bce}(G(H_t), A_t)$. For all deep learning methods, we tune the batch size $\{128, 256\}$, learning rate $\{5 \times 10^{-4}, 10^{-3}, 5 \times 10^{-3}\}$, hidden size $\{8, 16, 32\}$, and dropout rate 0, 0.1. Transformer-based models (DeepLTMLE, IGC-Net, and D^3-Net) additionally tune the number of layers $\{1, 2, 3\}$ and attention heads $\{2, 4\}$. DeepLTMLE and D^3-Net further tune $\alpha \in \{0.05, 0.1\}$, while IGC-Net additionally tunes the hidden representation size $\{8, 16, 32\}$.

D Semi-Synthetic Data DGP

DGP with Limited Time-varying Confounding. We adopt the semi-synthetic DGP from DeepACE. Using the preprocessing pipeline of Wang et al. [70], we extract 10 time-varying covariates $X_t \in \mathbb{R}^{10}$ over $\tau = 15$ time steps. These covariates are treated as observed patient states and are directly taken from real-world measurements.

Given the observed history, binary treatments $A_t \in \{0, 1\}$ are simulated according to a stochastic, dynamic treatment policy. The treatment assignment probability at time t depends on past covariates, past intermediate outcomes, and a treatment intensity variable $\ell_t = \ell_{t-1} + 2(A_t - 1)\bar{X}_t \tanh(Y_t)$, with initialization $\ell_0 = T/2 - 3$. Treatment is assigned as $\pi_t =$

$$\mathbb{1}\left\{\sigma\left(\sum_{i=1}^{h} \frac{(-1)^i}{1-i}\left(\bar{X}_{t-i} + \frac{\tanh(Y_{t-i})}{2}\right) - \tanh\left(\ell_{t-1} - \frac{T}{2}\right) + \epsilon_t^A\right) > 0.5\right\},$$

and outcomes are generated according to

$$Y_{t+1} = 5\sum_{i=1}^{h} \frac{(-1)^i}{1-i} \tanh\big(\sin(\bar{X}_{1:5,t-i}A_{t-i}) + \cos(\bar{X}_{5:10,t-i}A_{t-i})\big) + \epsilon_t^Y,$$

where $\bar{X}_{1:5,t-i}$ represent the mean of the first five covariates at step $t - i$, similarly for $\bar{X}_{5:10,t-i}$. The time lag $h = 8$. Noise terms satisfy $\epsilon_t^A, \epsilon_t^Y \sim \mathcal{N}(0, 0.5^2)$. Since our target estimand is the terminal outcome Y_τ, intermediate outcomes Y_t for $t < \tau$ are absorbed into L_{t+1}, yielding $L_t = (X_t, Y_{t-1})^\top \in \mathbb{R}^{11}$.

DGP with Expanded Time-varying Confounding . In the simple DGP above, patient covariates are unaffected by treatment. To introduce a stronger and more realistic treatment–confounder feedback loop, we further synthesize treatment-dependent time-varying covariates. Specifically, we generate latent covariates $Z_t \in \mathbb{R}^{d_z}$ with initialization $Z_1 \sim \mathcal{N}(0, I)$ and dynamics

$$Z_{t+1} = \omega_1 \cdot Z_t + \omega_2 \cdot A_t \sigma(Z_t^2) + \omega_3 \cdot 0.25 \tanh(\bar{X}_t) + \epsilon_t^Z,$$

where $\epsilon_t^Z \sim \mathcal{N}(0, 0.3^2)$ and the coefficients $\omega = (\omega_1, \omega_2, \omega_3)$ are randomly drawn and fixed to $(0.37, 0.42, 0.29)$. These synthesized covariates both affect and are affected by treatment.

Unless otherwise specified, we set ($d_z = 5$). To evaluate robustness in higher-dimensional settings, we additionally consider ($d_z = 10$) and ($d_z = 15$), generated using the same structural equations. We then concatenate Z_t with the observed covariates to form the augmented state $X_t^\dagger = (X_t, Z_t)^\top \in \mathbb{R}^{10+d_z}$.. Finally, treatment assignment and outcome generation follow the same structural equations as in the Limited Confounding DGP, with X_t replaced by $X_t^\dagger$. Noise distributions remain unchanged.
